# Improving Guideline Compliance and Documentation Through Auditing Neonatal Resuscitation

**DOI:** 10.3389/fped.2019.00294

**Published:** 2019-07-16

**Authors:** Laura Root, Henriette A. van Zanten, Maria C. den Boer, Elizabeth E. Foglia, Ruben S. G. M. Witlox, Arjan B. te Pas

**Affiliations:** ^1^Division of Neonatology, Department of Pediatrics, Leiden University Medical Center, Leiden, Netherlands; ^2^Division of Neonatology, Department of Pediatrics, University of Pennsylvania School of Medicine, Philadelphia, PA, United States

**Keywords:** neonate, newborn, neonatology, delivery room management, recordings

## Abstract

**Objective:** Evaluate whether weekly audits of neonatal resuscitation using video and physiological parameter recordings improved guideline compliance and documentation in medical records.

**Study design:** Neonatal care providers of the Neonatal Intensive Care Unit (NICU) of Leiden University Medical Center reviewed recordings of neonatal resuscitation during weekly plenary audits since 2014. In an observational pre-post cohort study, we studied a cohort of infants born before and after implementation of weekly audits. Video and physiological parameter recordings of infants needing resuscitation were analyzed. These recordings were compared with the prevailing resuscitation guideline and corresponding documentation in the medical record using a pre-set checklist.

**Results:** A total of 212 infants were included, 42 before and 170 after implementation of weekly audits, with a median (*IQR*) gestational age of 30 (27–35) weeks vs. 30 (29–33) weeks (*p* = 0.64) and birth weight of 1368 (998–1780) grams vs. 1420 (1097–1871) grams (*p* = 0.67). After weekly audits were implemented, providers complied more often to the guideline (63 vs. 77%; *p* < 0.001). Applying the correct respiratory support based on heart rate and respiration, air conditions (dry vs. humidified air), fraction of inspired oxygen (FiO_2_), timely start of interventions and evaluation of delivered care improved. Total number of correctly documented items in medical records increased from 39 to 65% (*p* < 0.001). Greatest improvements were achieved in documentation of present providers, mode of respiratory support and details about transport to the NICU.

**Conclusion:** Regular auditing using video and physiological parameter recordings of infants needing resuscitation at birth improved providers' compliance with resuscitation guideline and documentation in medical records.

## Introduction

The transition of a newly born is a complex event depending on several cardiopulmonary changes at birth. Guidelines containing step-by-step flowcharts on how to perform neonatal resuscitation are available for neonatal care providers to adequately provide care and improve outcome (1). To improve the quality of neonatal resuscitation, several Neonatal Intensive Care Units (NICUs) started recording and reviewing interventions in the delivery room (DR) on a regular basis (2–9). Reviewing vital physiological parameters and video imaging of neonatal resuscitation is reported to be a valuable audit tool for evaluating provided care (8) and can remove subjectivity (9–11).

We recently analyzed video and physiological parameter recordings of neonatal resuscitations performed at our unit. This study showed that providers often deviate from prevailing guidelines. Deviations mainly occurred within the first 30 s after birth. We speculated that these deviations were likely due to the stressful and often unpredictable circumstances during resuscitations (12). We also reported that documentation of DR management in the medical record was often incomplete and inaccurate, as compared to recordings (13). Our findings were in accordance with other studies observing guideline deviations (2, 14–16) or incomplete and inaccurate documentation of neonatal resuscitation (17–21). In order to improve DR management in our unit, we implemented weekly audits in 2014. The aim of this study was to assess whether weekly audits increased providers' compliance with the resuscitation guideline and improved documentation of DR management.

## Materials and Methods

A retrospective observational pre-post cohort study was performed at the NICU of Leiden University Medical Center (LUMC) in Leiden, the Netherlands, which is a tertiary level perinatal care center with an average of 850 intensive care admissions per year. During weekly audits, conducted since January 2014, we reviewed resuscitation recordings in plenary sessions. All NICU staff members were invited, but mostly neonatologists, fellows and residents attended audits. The audits took place in the morning, directly after handover, lasted about 15 min and were chaired by a coordinator who was also part of the NICU staff. During these audits, video recordings combined with respiratory function monitor recordings were used to evaluate delivered care in the DR. All providers were invited to provide feedback. Standard used evaluation criteria were hand hygiene, compliance to the prevailing resuscitation guideline, accuracy of documentation in the medical record, effect of interventions and lessons learned from the specific audit. During review of the recordings, providers received not only feedback on technical skills such as mask technique, but were also trained on cognitive skills such as physiology and clinical indications and behavioral skills such as communication.

We analyzed recordings of two cohorts of infants receiving support during transition after birth: before (2012–2013) and after (2016) the implementation of weekly audits. Recordings were compared with the prevailing local guideline for neonatal resuscitation and its documentation. Primary outcomes were total percentage of correctly performed and documented steps.

Plenary review of clinical performance can adversely affect providers. It is therefore important to design a review process that is acceptable to all participating providers. We excluded the years 2014 and 2015, because during this period the review process was adapted to the providers' needs and a culture of auditing as standard care was established.

For the purpose of this study, recordings were only used if they were complete and of good quality (e.g., no missing measurements, intact video file, infant visible on video recording). Recordings were analyzed by one independent investigator (LR) who was not part of the medical team when the audits were conducted. Before implementation of weekly audits there were two trolleys (one in the DR and one in the operation room) containing the equipment needed for recording and recordings were only performed if time for set up was sufficient. In the period after implementation, physiological parameters and videos were recorded using an all-in-one device available on all six resuscitation tables. The New Life Box (NLB Neo-RDS, Applied Biosignals, Weener, Germany) was used to record respiratory parameters (positive end-expiratory pressure (PEEP), peak inspiratory pressure (PIP), expired tidal volume and mask leak). Oxygenation and heart rate (HR) were measured using the Masimo SET pulse oximeter (Masimo Radical, Masimo Corporation, Irvine, California, USA), which was placed around the infant's right hand. Fraction of inspired oxygen (FiO_2_) was measured using a portable oxygen analyzer AX300-I (Teledyne Analytical Instruments, CA, USA), and applied pressure levels were registered by a variable orfice pneumometer (Avea Varflex Flow Transducer, Carefusion, Yorba Linda, CA, USA). All measurements were digitized and recorded at 200 Hz using the Bicore II (Cardinal Healthcare, Yorba Linda, CA) physiological recording system with Spectra physiological software (Grove Medical, London, UK) and Polybench software (Advanced Life Diagnostics, Weener, Germany).

The checklist for analysis of guideline compliance (Appendix a in Supplementary Material) was based on the same pre-set checklist used in our previous study (12). It contained observations and interventions needed to be performed according to our resuscitation guidelines, which are based on international and national guidelines. As in our previous study, we divided the guideline in step A, B and C. Step A contained all items “to be performed within 30 s after birth,” such as heat loss prevention (blanket or wrap), appropriate type of air condition (dry or humidified air) and correct start of FiO_2_ (21% or 30%). Step B contained items “to be performed within 1 min after birth,” such as the type of respiratory support given based on gestational age, HR and respiration. Step C contained items of cardiac resuscitation, to be performed “when the HR remained <60 beats per minute.” According to the guidelines, providers are supposed to evaluate HR, breathing and oxygen saturation directly after birth and assess the condition of the neonate every 30 s to evaluate the effect of an intervention and determine further interventions.

Documentation of DR management was done retrospectively by one of the providers performing the resuscitation and was stored in Patient Data Management System (PDMS) (Metavision; IMDsoft, Tel Aviv, Israel), a digital medical record. The documentation was compared with the recordings, using the same checklist as in our previous study (Appendix b in Supplementary Material) (13). Analyzed documentation included notes of resuscitation reports divided in initial evaluation, respiratory support, oxygen therapy, intubation, cardiac resuscitation, transport to NICU and subjectivity of documentation. Items were compared to the recordings and scored as either correctly or incorrectly documented, or correctly or incorrectly not documented. Some items could not be observed in the video recordings, such as umbilical cord pH, time of birth or time of transport to NICU. These items were only scored as being documented or not documented in the medical record. Baseline characteristics, such as year of birth, gestational age, gender, birth weight, mode of delivery, multiple birth and Apgar score (AS), were as well derived from PDMS.

All statistical analyses were performed using SPSS (SPSS for Windows, version 23, IBM, Chicago, IL, USA). Results are presented using the median (interquartile range (IQR)) or percentages. Chi-square tests were performed on categorical data. Mann Whitney *U*-tests were used for comparison of numerical data. A *p*-value < 0.05 was considered statistically significant.

## Results

A total of 382 recordings of neonatal resuscitation were available. Recordings of 170 infants were excluded due to low quality of the recordings. The remaining recordings of 212 infants were analyzed: 42 recordings from infants born before and 170 from infants born after implementation of weekly audits. Baseline characteristics did not differ between cohorts with a median (*IQR*) gestational age of 30 (27–35) weeks vs. 30 (29–33) weeks (*p* = 0.64) and birth weight of 1368 (998–1780) grams vs. 1420 (1097–1871) grams (*p* = 0.67) (Table 1).

**Table 1:** Baseline characteristics.

	**2012–2013 (*n* = 42)**	**2016 (*n* = 170)**	***p*-value**
Male	25 (60)	97 (57)	0.77^a^
Preterm (<32 weeks)	26 (62)	120 (70)	0.28^a^
Gestational age (in weeks)	30 (27–35)	30 (29-33)	0.64^b^
Birth weight (in grams)	1368 (998–1780)	1420 (1097-1871)	0.67^b^
Born by cesarean section	22 (52)	104 (61)	0.30^a^
Multiple birth	11 (26)	62 (36)	0.21^a^
AS documented			
1 min	6 (4–7)	6 (3-8)	0.90^b^
5 min	8 (6-9)	8 (7-9)	0.41^b^
10 min	9 (7-9)	9 (8-9)	0.32^b^

*Data are presented as n (%) or median (IQR)*.

a *Chi-Squared test*.

b *Mann-Whitney U-test*.

### Compliance With Guideline

After implementation of weekly audits, guideline compliance improved: the overall number of correctly performed steps increased from 63 to 77% (*p* < 0.001).

#### Step A

Timely initial evaluation and interventions according to the guideline in step A increased significantly (before vs. after implementation of weekly audits: 58 vs. 72%; *p* < 0.001). After implementation, all infants received appropriate air condition (dry or humidified air) during respiratory support (74 vs. 100%; *p* < 0.001), and providers started with the correct FiO_2_ (58% vs. 97%; *p* < 0.001) (Table 2).

**Table 2:** Compliance with resuscitation guideline.

	**2012–2013 (*n* = 42)**	**2016 (*n* = 170)**	***p*-value**
**STEP A “WITHIN 30 S AFTER BIRTH”**			
Time of arrival on resuscitation table (s)	37 (25–60)	33 (19–52)	0.30^b^
<30 s	12 (32)	63 (45)	0.13^a^
Time from arrival to wearing a hat (s)	8 (3–14)	8 (4–13)	0.98^b^
<30 s	7 (20)	38 (32)	0.18^a^
Correctly blanket or wrap	42 (100)	168 (99)	1.00^c^
Appropriate type of air condition	31 (74)	170 (100)	<0.001^c^
Auscultation HR performed	36 (86)	147 (86)	0.98^a^
Time from arrival to auscultation (s)	32 (19–56)	37 (20–66)	0.47^b^
<30 s	2 (6)	23 (16)	0.17^c^
Duration HR evaluation (s)	12 (5–20)	13 (8–22)	0.19^b^
>5 s	27 (75)	123 (84)	0.19^a^
Correct start of FiO_2_	14 (58)	135 (97)	<0.001^c^
**STEP B “WITHIN 1 MIN AFTER BIRTH”**			
Time from arrival to start support (s)	34 (19–74)	16 (10–26)	<0.001^b^
<1 min after birth	10 (28)	98 (70)	<0.001^a^
Correct start PEEP (cm H2O)	35 (95)	134 (89)	0.53^c^
Correct first step (type of respiratory support given)	29 (73)	144 (89)	0.006^a^
Correct pressure 1st sustained inflation (cm H2O)	24 (82)	100 (88)	0.37^c^
Correct duration 1st sustained inflation (s)	12 (43)	60 (58)	0.15^a^
2nd evaluation performed	19 (68)	101 (99)	<0.001^a^
Correct duration 2nd evaluation (s)	13 (46)	85 (83)	<0.001^a^
Correct 2nd step (type of respiratory support given)	20 (71)	101 (98)	<0.001^c^
Correct pressure 2nd step (cm H2O)	18 (78)	54 (73)	0.61^a^
Correct duration 2nd sustained inflation (s)	-	29 (43)	-
3rd evaluation performed	20 (87)	67 (93)	0.39^a^
Correct duration 3rd evaluation (s)	19 (83)	50 (69)	0.20^a^

*Data are presented as n (%) or median (IQR)*.

a *Chi-Squared test*.

b *Mann-Whitney U-test*.

c *Fisher's exact test*.

#### Step B

Most significant improvements were made in step B (before vs. after implementation of weekly audits: 68 vs. 83%; *p* < 0.001). Improved items included start of appropriate respiratory support (28 vs. 70%; *p* < 0.001), as well as applying the correct respiratory support based on HR and respiration (73 vs. 89%; *p* = 0.006) (Table 2).

#### Step C

Two infants received chest compressions, both after implementation of weekly audits. Indication and technique were both correct, as the combination of ventilation vs. compressions was 1:3 and chest compressions were performed with 2 thumbs or 2 fingers.

### Accuracy of Documentation

After implementation of weekly audits, accuracy of documentation of DR management improved significantly (before vs. after implementation of weekly audits: 39 vs. 65%; *p* < 0.001).

#### Initial Evaluation

Documentation improved in the following items: providers present at resuscitation (2 vs. 82%; *p* < 0.001), physical condition of infants upon arrival on the resuscitation table (69 vs. 95%; *p* < 0.001), quality of breathing at first impression (60 vs. 91%; *p* < 0.001), and documentation of umbilical cord blood gas samples (43 vs. 68%; *p* = 0.002) (Table 3).

**Table 3:** Accuracy of documentation.

	**2012–2013 (*n* = 42)**	**2016 (*n* = 170)**	***p*-value**
**GENERAL**			
Time of birth	40 (95)	167 (98)	0.26^a^
Umbilical cord pH	18 (43)	116 (68)	0.002^a^
Providers present	1 (2)	139 (82)	<0.001^a^
**INITIAL EVALUATION AT BIRTH**			
Clinical condition	29 (69)	161 (95)	<0.001^a^
HR	20 (48)	101 (59)	0.17^a^
Oxygen saturation	4 (10)	30 (18)	0.20^a^
Quality of breathing	25 (60)	155 (91)	<0.001^a^
**RESPIRATORY SUPPORT**			
Type of 1st respiratory support given	35 (95)	148 (99)	0.18^b^
Starting time respiratory support	5 (14)	45 (30)	0.04^a^
Indication respiratory support	15 (41)	69 (46)	0.55^a^
Sustained inflations (SI) given	26 (79)	102 (94)	0.02^b^
Consecutive inflations (PPV) given	18 (60)	74 (80)	0.02^a^
Ventilation pressures used	12 (32)	116 (77)	<0.001^a^
Ventilation pressures adjusted	10 (29)	120 (86)	<0.001^a^
Effect on HR	11 (30)	83 (55)	0.005^a^
Effect on oxygen saturation	14 (38)	123 (82)	<0.001^a^
Duration of respiratory support	2 (5)	7 (5)	1.00^b^
**OXYGEN THERAPY**			
Use of supplemental oxygen	17 (68)	139 (98)	<0.001^b^
Starting time of oxygen therapy	1 (4)	21 (15)	0.20^a^
Indication for oxygen therapy	3 (12)	50 (35)	0.02^a^
Starting point of oxygen therapy	3 (12)	73 (51)	<0.001^a^
Maximum level of FiO_2_	9 (36)	117 (82)	<0.001^b^
Effect of supplemental oxygen	7 (28)	110 (78)	<0.001^a^
Duration of oxygen therapy	1 (4)	5 (4)	1.00^b^
FiO_2_ titrated during resuscitation	10 (40)	122 (86)	<0.001^a^
**TRANSPORT TO NICU**			
Time of transport to NICU	1 (2)	19 (11)	0.14^a^
Type of support during transport	21 (66)	130 (88)	0.002^a^
Ventilation pressures during transport	10 (31)	116 (78)	<0.001^a^
FiO_2_ during transport	13 (41)	121 (82)	<0.001^a^

*Data are presented as n (%)*.

a *Chi-Squared test*.

b *Fisher's exact test*.

#### Respiratory Support

Significant improvements in documentation included starting time of respiratory support (14 vs. 30%; *p* = 0.04), whether sustained inflations (SI) were given (79 vs. 94%; *p* = 0.02) and ventilation pressures (32 vs. 77%; *p* < 0.001) (Table 3).

#### Oxygen Therapy

FiO_2_ was recorded in 25/42 infants before implementation and in 146/170 infants after implementation of weekly audits. Documentation of used supplemental oxygen (68 vs. 98%; *p* < 0.001), indication for oxygen therapy (12 vs. 35%; *p* = 0.02) and effect of supplemental oxygen (28 vs. 78%; *p* < 0.001) increased (Table 3).

#### Intubation

Intubation occurred in 4/42 infants before and 14/170 infants after implementation of weekly audits, which was correctly reported in both cohorts. Documenting the indication for intubation improved (25 vs. 86%; *p* = 0.04). However, not all details were always reported, including the tube size (75 vs. 86%; *p* = 1.00), fixation depth (50 vs. 50%; *p* = 1.00), and the number of intubation attempts (25 vs. 57%; *p* = 0.58).

#### Cardiac Resuscitation

Two infants received cardiac resuscitation after implementation of weekly audits. Indication and starting time of cardiac resuscitation was missing for one infant; duration was not reported for both.

#### Transport to NICU

Documentation about transport to the NICU improved significantly (before vs. after implementation of weekly audits). Improved items included type of respiratory support (66 vs. 88%; *p* = 0.002), ventilator settings (31 vs. 78%; *p* < 0.001), and FiO_2_ (41 vs. 82%; *p* < 0.001) (Table 3).

#### Subjectivity of Documentation

Subjective medical terms such as “low heart rate” and “pink” were frequently found in documentation (before vs. after implementation of weekly audits: 62 vs. 57%; *p* = 0.57). No non-medical terms (e.g., “looking bad”) were used.

Documented AS were compared with the condition of the infant as observed in the recordings. When AS could be scored, similarity in AS was found in 45 vs. 64% (*p* = 0.04) for AS 1 min, 61 vs. 74% (*p* = 0.004) for AS 5 min, and 88 vs. 80% (*p* = 0.14) for AS 10 min.

## Discussion

This study showed that weekly audits, using video and physiological parameter recordings of infants receiving support during neonatal transition, improved guideline compliance and documentation in medical records.

Worldwide, several NICUs reported systematic recording of neonatal resuscitation for quality improvement (2–9). Benefits of recording real-time healthcare delivery for quality improvement purposes were reported (22), but previous studies failed to prove a significant improvement of overall clinical performance after reviewing recordings of neonatal resuscitation (7, 14, 23, 24). A recent study showed that practice PPV (positive pressure ventilation) psychomotor training combined with team debriefings using video recordings of actual resuscitations may improve time to effective spontaneous breathing and adherence to guidelines during real neonatal resuscitations (25), but this study did not analyze complete neonatal resuscitation. To the best of our knowledge, our study is the first to prove significant improvement in overall guideline compliance and documentation of neonatal resuscitation after weekly plenary auditing. The International Liaison Committee on Resuscitation (ILCOR) (1) highlights the importance of good quality of neonatal resuscitation in their guideline. We therefore postulate that improved guideline compliance results in improved neonatal resuscitation. A recent study showed that guideline compliance improved clinical outcome after adult resuscitation (26). This may also apply to neonatal resuscitation, suggesting that weekly audits furthermore contributes to better patient outcomes.

Improvement in guideline compliance and documentation may be due to various factors. Studies reported that knowledge and skills quickly diminish after neonatal resuscitation training (27, 28), hence boosting personal level of knowledge and skills is highly recommended (29). Our weekly audits allow providers to continuously train, refresh knowledge, and learn from others. Although the resuscitation guideline changed during the study period, we still observed overall better guideline compliance, suggesting that regular auditing contributes to internalizing the prevailing guideline.

Reviewing recordings of neonatal resuscitation enables valuable discussions on subjectivity in the guideline and appropriateness of interventions during neonatal resuscitation for individual patients. Clinical parameters such as HR, chest excursions and color can be difficult to interpret as they are very subjective and subtle (30, 31). The use of electrocardiogram, pulse oximetry, and a respiratory function monitor can add objectivity to clinical assessment (9). Respiratory function monitor data, including ventilation pressures, mask leak, airway obstruction, and breathing pattern, can be discussed during audits, which results in objective feedback about the appropriateness of interventions (9).

Although documentation is the golden standard, documentation is almost never fully accurate. Accurate documentation enables reliable evaluation of resuscitation practice and legal review of medical records and ensures quality of post resuscitation care (13). Parameter recordings can add accuracy, as these can provide detailed information about the resuscitation process (9–11). Parameter recordings may therefore be a useful tool for completing documentation.

Auditing neonatal resuscitation allows providers to mutually provide feedback. Earlier we reported that providers would recommend other NICUs to implement audits, assuming that preconditions for a safe learning environment are met (32). Providers emphasized that audits should be blame and shame free, non-punitive, and focused on educational benefits, amongst others. Meeting preconditions for a safe learning environment allows providers to freely elaborate on their performances, which improves discussions during the audits.

Some NICUs that already implemented recording and reviewing neonatal resuscitation reported concerns about the legal and ethical implications of recording and auditing neonatal resuscitation and possible negative impact on providers (8, 33, 34). These factors should be considered carefully upon implementation. When preconditions for a safe environment are met, we recommend other NICUs to record and review neonatal resuscitation on a regular basis.

This study is the first to investigate the influence of weekly audits on providers' guideline compliance and accuracy of documentation. However, it cannot simply be assumed that the overall improvement in guideline compliance (63 vs. 77%) and accuracy of documentation (39 vs. 65%) is fully effectuated by the audits and that this improvement is of clinical relevance, although our recent study showed that providers do experience recording and reviewing neonatal resuscitation as highly valuable for maintaining and improving resuscitation skills (32). Improvement in guideline compliance and documentation may also be caused by the awareness of being observed. This has been reported to improve the outcome in various studies (22, 35), and may apply specifically to rounding residents.

Our study does have some limitations. Due to convenience sampling in this retrospective pre-post cohort study we could not perform a power analysis. No observer bias occurred during analysis of the recordings, as only one researcher scored all data, but there is potential bias as the video reviewer was not blinded to the study phase. It is standard practice that video recording is simultaneously used with physiological parameters in our unit, however some recordings could not be used in our study due to an error in the video or measurement files, the infant being not visible on video due to camera angle, or missing documentation, amongst others. Pre- and post-implementation numbers of cases are not balanced as in the period before implementation of weekly audits there were only two trolleys containing the equipment needed for recording, and recordings were only performed if time for set up was sufficient. In the period after implementation, physiological parameters and video were recorded using an all-in-one device available on six resuscitation tables, but technical problems with recordings sometimes persisted. Furthermore, we adapted our review process to providers' needs and we met preconditions for a safe environment. Therefore, we presume that it is unlikely that selection bias occurred during this study.

To score guideline compliance and accuracy of documentation we used the same pre-set checklists as in our previous studies (12, 13), which have not been externally validated. In order to increase validity in scoring guideline compliance it would be good to design an evidence based model to assess quality of neonatal resuscitation. Furthermore, including a guideline for documentation may improve accuracy, knowledge and in-depth understanding of neonatal resuscitation processes. As a result of weekly audits, documentation of neonatal resuscitation became more accurate. Discussions about documentation allowed providers to identify items crucial to report in medical records. For instance, documenting time until cord clamping could have given providers valuable insights in the efficiency of logistics directly after birth. More research is needed in order to decide on what items should be included in standard documentation.

Our weekly audits may still be optimized. For instance, adding audio to the recordings and inviting nurses during audits may improve the quality of delivered care even further. Furthermore, although we showed the effect of auditing on a weekly basis, it is unclear whether this effect would have been the same for auditing with another frequency. Further research is needed in order to determine the optimal frequency of audits, as frequently recurring short-lasting booster sessions, adjusted to the specific situation of a NICU, may allow for better integration in the daily routine of NICUs and extra exposure for providers (32).

## Conclusion

Regular auditing using video and physiological parameter recordings of infants needing resuscitation at birth improved providers' compliance with resuscitation guideline and documentation in medical records. When preconditions for a safe environment are met, regular auditing can be recommended.

## Data Availability

The datasets generated for this study are available on request to the corresponding author.

## Ethics Statement

Recording video simultaneously with physiological parameters of neonatal resuscitation is considered standard care in the LUMC since 2016 and recordings thus became part of the medical record. In the Netherlands, no ethical approval is required for studies with anonymized data from medical records and for patient data collected during standard care. The LUMC Medical Ethics Committee provided a statement of no objection for conducting this study.

## Author Contributions

LR performed the literature search, data collection, interpretation and analysis, and wrote the manuscript. AtP conceptualized and designed the study, and reviewed, revised, and approved the manuscript. HvZ, MdB, EF, and RW critically reviewed the data analyses, and critically reviewed and approved the final manuscript for publication. All authors agree to be accountable for all aspects of the work.

### Conflict of Interest Statement

The authors declare that the research was conducted in the absence of any commercial or financial relationships that could be construed as a potential conflict of interest.

## Abbreviations

DR: delivery room
NICU: Neonatal Intensive Care Unit
FiO_2_: fraction of inspired oxygen
PEEP: positive end-expiratory pressure
PPV: positive pressure ventilation
HR: heart rate
AS: Apgar score.
