# Supplementary material for: Improving Guideline Compliance and Documentation Through Auditing Neonatal Resuscitation
Source: Front Pediatr. 2019 Jul 16;7:294. doi: 10.3389/fped.2019.00294 (PMC6646726; doi:10.3389/fped.2019.00294)
Supplement: Supplementary file 1 [file Data_Sheet_1.docx]

| **Step A ‘to be performed within 30 s from birth’**   - Time of arrival on resuscitation table (s)   Arrival < 30 s on resuscitation table   - Heat loss prevention   Time from arrival to wearing a hat (s)  Wearing a hat < 30 s  Correctly blanket or wrap   - Appropriate type of air condition (dry or humidified air) - Auscultation HR performed   Time from arrival on resuscitation table to auscultation (s)  Auscultation within 30 s from birth  Duration HR evaluation (s)  HR evaluation > 5 s   - Correct start of FiO_2_ (21 or 30%) |
| --- |
| **Step B ‘to be performed within 1 min after birth’**   - Time from arrival to start respiratory support (s)   Start support within 1 min after birth   - Correct start PEEP (cm H2O) - Correct first step (type of respiratory support given)   Correct pressure first sustained inflation (cm H2O)  Correct duration first sustained inflation (s)   - 2^nd^ evaluation performed   Correct duration 2^nd^ evaluation (s)   - Correct 2^nd^ step (type of respiratory support given)   Correct pressure 2^nd^ step (cm H2O)  Correct duration 2^nd^ sustained inflation (s)   - 3^rd^ evaluation performed   Correct duration 3^rd^ evaluation (s) |
| **Step C ‘when HR < 60 beats per minute’**   - Indication chest compressions - Technique chest compressions - Combination ventilation:compressions 1:3 - Compressing the chest with two thumbs or two fingers |

**Appendix a: Checklist scored items resuscitation guideline**

| **General**   - Time of birth - Umbilical cord pH - Providers present   **Initial evaluation at birth**   - Clinical condition - HR - Oxygen saturation - Quality of breathing |
| --- |
| **Respiratory support**   - Type of 1^st^ respiratory support given - Starting time respiratory support - Indication for respiratory support - Sustained inflations (SI) given - Consecutive inflations (PPV) given - Ventilation pressures used - Ventilation pressures adjusted - Effect of respiratory support on HR - Effect of respiratory support on oxygen saturation - Duration of respiratory support |
| **Oxygen therapy**   - Use of supplemental oxygen - Starting time of oxygen therapy - Indication for oxygen therapy - Starting point of oxygen therapy - Maximum level of FiO_2_ - Effect of supplemental oxygen - Duration of oxygen therapy - FiO_2_ titrated during resuscitation |
| **Intubation**   - Indication for intubation - Tube size - Fixation depth tube - Number of tries before successful intubation - Difficulty of intubation mentioned |
| **Cardiac resuscitation**   - Indication for chest compressions - Starting time of chest compressions - Duration of chest compressions |
| **Transport to NICU**   - Time of transport to NICU - Type of respiratory support during transport - Ventilation pressures during transport - FiO_2_ during transport |
| **Quality of documentation**   - Use of subjective language versus objective language in medical record - Use of medical terms versus non-medical terms in medical record - AS given by caregiver versus researcher after reviewing video recordings |
| *Items were scored as either correctly or incorrectly documented. In case of correctly documented, there were two options: (1) the event occurred and was documented (‘correctly documented’) or (2) the event did not happen and was correctly not documented (‘correctly not documented’), when confirmed by the recording. In case of incorrectly documented, there were three options: (1) not documented but observed on the recording (‘incorrectly not documented’), (2) documented but not confirmed on the recording (‘incorrectly documented’) or (3) documented but with incorrect time indication.* |

**Appendix b: Checklist scored items documentation of DR management**
